# Effects of Probiotics on Cognitive Reactivity, Mood, and Sleep Quality

**DOI:** 10.3389/fpsyt.2019.00164

**Published:** 2019-03-27

**Authors:** Angela Marotta, Eleonora Sarno, Antonio Del Casale, Marco Pane, Luca Mogna, Angela Amoruso, Giovanna E. Felis, Mirta Fiorio

**Affiliations:** ^1^Department of Neurosciences, Biomedicine and Movement Sciences, University of Verona, Verona, Italy; ^2^Neurology Unit, Neuroscience Department, Azienda Ospedaliera Universitaria Integrata, Verona, Italy; ^3^Department of Biotechnology, University of Verona, Verona, Italy; ^4^Open Innovation Department, Microbion SRL, Verona, Italy; ^5^BIOLAB RESEARCH SRL, Novara, Italy

**Keywords:** psychological well-being, probiotics, mood, sleep quality, personality traits

## Abstract

Recent demonstration that probiotics administration has positive effects on mood state in healthy populations suggests its possible role as an adjunctive therapy for depression in clinical populations and as a non-invasive strategy to prevent depressive mood state in healthy individuals. The present study extends current knowledge on the beneficial effects of probiotics on psychological well-being, as measured by changes in mood (e.g., cognitive reactivity to sad mood, depression, and anxiety), personality dimensions, and quality of sleep, which have been considered as related to mood. For this double-blind, placebo-controlled study 38 healthy volunteers assigned to an experimental or control group assumed a daily dose of a probiotic mixture (containing *Lactobacillus fermentum* LF16, *L. rhamnosus* LR06, *L. plantarum* LP01, and *Bifidobacterium longum* BL04) or placebo, respectively, for 6 weeks. Mood, personality dimensions, and sleep quality were assessed four times (before the beginning of the study, at 3 and 6 weeks, and at 3 weeks of washout). A significant improvement in mood was observed in the experimental group, with a reduction in depressive mood state, anger, and fatigue, and an improvement in sleep quality. No between-groups differences were found. These findings corroborate the positive effect of probiotics on mood state and suggest that probiotics administration may improve psychological well-being by ameliorating aspects of mood and sleep quality.

## Introduction

Gut and brain communicate with each other via the gut-brain axis, a specific pathway that involves the neural, endocrine, and immune systems (1–4). The intestinal microbiota plays a crucial role in this bidirectional communication, since it can influence mood and cognitive functions by producing neurotransmitter precursors that reach the brain through the endocrine and the autonomic nervous systems where they regulate the level of specific neurotransmitters (5, 6).

It has been hypothesized that modifying the microbial environment by means of probiotics, for instance, may help to improve mood and cognitive functions. This hypothesis is supported by studies using animal models. Li et al. (7) found an improvement in spatial memory and problem solving in rats after probiotics supplementation. Other studies using rodent models showed beneficial effects of probiotics on anxiety and depression (8, 9). Research involving human participants is still scant. Among the existing literature on this topic, some studies showed no effects of probiotics on mood (10), but other found promising data. For instance, a recent fMRI study demonstrated that 4 weeks of probiotic intake reduced the activity of brain regions involved in emotional processing (11). Moreover, a recent triple blind, randomized, placebo-controlled study showed reduced cognitive reactivity to sad mood after a 4-week intervention with multispecies probiotics (12). Sleep quality also seems to benefit from probiotic supplementation. Takada et al. (13) reported an improvement in sleep quality in academic students during a period of increasing stress after 11 weeks of probiotics consumption. Beneficial effects of probiotics were also reported in studies involving clinical populations. For instance, Rao et al. (14) found that anxiety in patients with chronic fatigue syndrome was reduced after 2 months of probiotics intake. Overall, these studies suggest that probiotics administration might be used to improve mood and cognitive functions (e.g., emotional processing).

The aim of the present explorative study was to extend our current knowledge about the beneficial effects of probiotics on psychological well-being by investigating different aspects of mood (e.g., cognitive reactivity to sad mood, depression, and anxiety) and various dimensions of personality related to mood, including optimism, pessimism, and motivational drive. Also, we investigated the potential effects of probiotics on sleep quality, which is also known to affect mood (15). In line with previous findings, we expected an improvement in mood only in the group that received the probiotics. Additionally, we hypothesized an improvement in perceived sleep quality and personality dimensions known to be related to anxiety and depression.

Examining the ways in which probiotics influence mood, personality traits, and sleep may help to gain a better understanding of the relationship between the gut and the brain and to develop new applications for psychological well-being.

## Materials and Methods

Participants were recruited from students of the University of Verona (Italy) where the investigation took place and where data were collected. Computation of the sample size was performed with G-Power 3.1 (16), considering *F*-test within-between interaction with two groups (experimental and control) and four evaluation sessions (T0, T1, T2, T3) (see the procedure for details). Assuming a priori medium effect size of 0.25 (17), an α error probability of 0.05, and a power (1-β error probability) of 0.90, the resulting total sample size is 30. We recruited more participants to prevent reduction in statistical power due to potential drop-outs.

Thirty-eight male and female adults volunteered for the study (13 women, mean age ± SD, 22.00 ± 3.02, age range, 19–33 years). Inclusion criterion was an age between 18 and 35 years. Exclusion criteria were: psychiatric or neurological disorders, celiac disease, lactose intolerance, or allergies or other ongoing illnesses (i.e., irritable bowel syndrome, diabetes, ulcerative colitis, etc.) or recent antibiotic treatment (i.e., <3 months before the beginning of the study). Participants who smoked more than 10 cigarettes per day were excluded.

Participants were screened via structured interviews by three experimenters (MF, AM, ES) who collected data on demographics and physical activity. In order to account for potential bias due to premenstrual syndrome, the women were also asked about their monthly physical and emotional changes.

Participants were assigned to receive probiotics or placebo by means of stratified randomization method. Since the amount of physical activity can influence cognitive functions, mood and sleep quality (18, 19), we took this covariate into account in the randomization process. More precisely, we applied the stratified randomization method to address the need of balancing the groups for the amount of physical activity (20, 21). Following this method, participants were stratified into two blocks (Highs and Lows) of 19 participants each depending on the amount of physical activity. More precisely, according to the mean value of physical activity per week of the whole sample (2.87 ± 0.99 h), the block of Highs was made of participants with more than 2.87 h per week and the block of Lows was made of participants with <2.87 h per week). Then, participants were allocated to the experimental or control group by means of simple randomization through a computer generated list that guarantees the same number of subjects and equivalent physical activity by type of intervention. In this way, sample size and amount of physical activity were balanced in the experimental and control group. After randomization, we confirmed that the level of physical activity was balanced between the two groups [*t*_(34)_ = 1.031, *p* = 0.310]. Two experimenters (MA and ES) enrolled participants, generated the random allocation sequence, and assigned participants to interventions. The experimenters and the participants were blinded for group allocation.

The study was conducted according to the principles expressed in the Declaration of Helsinki and was approved by the Ethical committee of Verona Hospital (Azienda Ospedaliera Universitaria Intregrata, AOUI Verona, 766CESC). Written informed consent was obtained before the start of the study. The protocol is registered in ClinicalTrials.gov with the number ID: NCT03539263. The full trial protocol can be accessed upon request at Probiotical S.p.A. and to the principal investigator MF.

### Procedure

The probiotic product or placebo was administered for 6 weeks followed by a 3-week washout period for a total duration of 9 weeks. The experimental group received 42 sachets of the product (one for each day), each containing 4 × 10^9^ colony forming unit/active fluorescent unit (CFU/AFU) 2.5 g freeze-dried powder of the probiotic mixture containing *Lactobacillus fermentum* LF16 (DSM 26956), *L. rhamnosus* LR06 (DSM 21981), *L. plantarum* LP01 (LMG P-21021), and *Bifidobacterium longum* BL04 (DSM 23233) (Probiotical S.p.A., Novara, Italy).

The mixture was composed with commercially available probiotic strains extensively used for food supplement formulations.

The study materials were analyzed by Biolab Research S.r.l., Novara, Italy, via flow cytometry (ISO 19344:2015 IDF 232:2015, > 4 × 10^9^ AFU) and plate count method (Biolab Research Method 014-06, >4 × 10^9^ CFU) to confirm target cell count. The control group received 42 sachets of placebo, each containing 2.5 g of maltodextrin in powder form. The placebo powder was indistinguishable from the probiotics powder in color, taste, and smell, but contained no probiotic bacteria. Participants were instructed to dissolve the powder in water or milk and drink it in the morning with breakfast.

A mobile application developed *ad hoc* (AgendaQR, Bussola Labs, Verona, Italy) was installed on the participants' phones at the beginning of the study; adherence to the study protocol was facilitated by means of a daily reminder to take the probiotics product or placebo and to scan the QR code attached to the sachet. If a participant failed to scan the code, the application kept a record of the missed daily intake.

All participants completed an extensive battery of psychological questionnaires (see next paragraph for a details) at four time points: before intake of the probiotics product or placebo (baseline) (T0), at 3 (T1), and 6 (T2) weeks after the first intake, and then at 3 weeks of washout (T3). E-Prime 2.0 software (Psychology Software Tools Inc., Sharpsburg, PA, USA) was used to present the questionnaires and record the responses. Each session lasted about 45 min.

### Questionnaires Measuring Mood-Related Aspects

#### Leiden Index of Depression Sensitivity-Revised Test

The primary outcome of our study was the difference in the scores at the Leiden Index of Depression Sensitivity-Revised test (LEIDS-R) (22) between the experimental and control groups. The LEIDS-R is a self-report questionnaire that tests cognitive reactivity to sad mood, which is an index of cognitive vulnerability to depression. It consists of 34 items describing different situations. Before answering the items, participants are asked to take a few minutes to imagine their feelings and thoughts when they experience a sad mood. They then rate how much each item applies to themselves on a 5-point scale ranging from 0 (*not at all*) to 4 (*very strongly*). Of note, the experimenter emphasizes that each item describes a situation happening on *a day that is not good, but you don't feel depressed*. The LEIDS-R consists of 6 subscales: Hopelessness/Suicidality (5 items), Acceptance/Coping (6 items), Aggression (5 items), Control/Perfectionism (6 items), Risk aversion (6 items), and Rumination (6 items). The total score for each subscale is obtained by adding the scores from the corresponding item. The range of the total score for the Aggression and Hopelessness/Suicidality subscales is from 0 to 20. The range of the total score for the other three scales is from 0 to 24. The higher the total subscale score, the higher the vulnerability to the assessed dimension.

#### State Trait Anxiety Inventory

The State Trait Anxiety Inventory, STAI, (23) is a self-report questionnaire that measures the presence and severity of current symptoms of anxiety and the propensity to be anxious (24). There are separate subscales for two anxiety components: state anxiety (STAI—form Y1) and trait anxiety (STAI—form Y2). Each subscale contains 20 items. The STAI—Y1 measures the current anxiety state by asking participants to rate how they actually feel (e.g., calm, tense, worried) on an intensity scale from 1 (*not at all*) to 4 (*very much so*). The STAI—Y2 measures the propensity to anxiety by asking participants to rate how they generally feel in their life (e.g., confident) on a frequency scale from 1 (*almost never*) to 4 (*almost always*). The total score for each subscale is obtained by adding the scores of all items. The score for the anxiety-absent items are reversed. The range of the total score for each subscale is 20–80, wherein the higher the score, the higher the degree of state or trait anxiety. Scores of 39–40 have been defined as a cut-off for clinically relevant symptoms of state anxiety (24).

#### Beck Depression Inventory

The Beck Depression Inventory, BDI-2 (25), is a self-report questionnaire that measures the occurrence and severity of current depressive symptoms. It consists of 21 groups of sentences describing different depression-related feelings and thoughts (e.g., self-dislike, loss of interest, irritability, changes in sleep pattern). Participants are asked to choose the statement from a group of sentences that best describes how they have been feeling in the past 2 weeks including the current day. Each statement corresponds to a specific severity score (range, 0–3). The total score is obtained by adding the statement scores (range, 0–63), wherein the higher the total score, the higher the severity of depressive state (minimal depression, 0–13; mild depression, 14–19; moderate depression, 20–28; severe depression, 29–63).

#### Profile of Mood State

The Profile of Mood State, POMS, (26) is a self-report questionnaire that assesses mood. It consists of 58 items (words or sentences) that describe feelings that people usually have. For each item, participants rate on a 5-point scale from 0 (*not at all*) to 4 (*extremely*) how they have been feeling the past week including the current day. The items are grouped in 6 subscales: Tension (9 items), Depression (15 items), Anger (12 items), Fatigue (7 items), Confusion (7 items), and Vigor (8 items). The Vigor scale is in inverted relationship with the other scales since it assesses positive feelings (e.g., to be lively, active, energetic). The total score for each scale is obtained by adding the related item, wherein the higher the total score of the scale, the higher the level of the related mood.

### Sleep Quality Questionnaire

#### Pittsburgh Sleep Quality Index

The Pittsburgh Sleep Quality Index, PSQI, (27, 28) is a self-report questionnaire that assesses sleep quality for the majority of days and nights in the past months. It consists of 19 questions. The first four investigate usual bedtime, the number of minutes needed to fall asleep, the usual getting up time, and the hours of sleep per night. The other questions are related to other aspects of sleep quality rated on scales appropriate for the specific question. The items are weighted on a 0–3 interval scale and grouped in seven component scores (i.e., subjective sleep quality, sleep latency, sleep duration, use of sleeping medication, daytime dysfunction, sleep duration, habitual sleep efficiency). A global PSQI score (range, 0–21) is obtained by adding up the component scores, wherein the lower the PSQI global score, the better the sleep quality.

### Questionnaires Measuring Personality-Related Aspects

#### Temperament and Character Inventory

The Temperament and Character Inventory, TCI, (29) is a self-report, true-false questionnaire that measures two components of personality: temperament and character. Temperament refers to automatic responses to perceptual stimuli likely reflecting hereditable biases in information processing (29). It consists of four dimensions: novelty seeking (system of behavioral activation), harm avoidance (system of behavioral inhibition), reward dependence (system for maintenance of ongoing behavior), and persistence (system of partial reinforcement and active behavior despite fatigue and frustration). Based on the psychobiological model of personality (29), each dimension is related to specific neurotransmitters: dopamine for novelty seeking; GABA and serotonin for harm avoidance; noradrenaline and serotonin for reward dependence, and glutamate and serotonin for persistence. Character refers to dimensions determined by the environment rather than by inheritance. These dimensions regulate the cognitive processes of perception and emotion defined by the person's temperament (30). The character dimensions are: self-directedness (identification with autonomous self and ability to solve situations); cooperativeness (extent to which other people are viewed as part of the self); and self-transcendence (identification with a unity of all things). Based on this model of personality, the TCI consists of 240 items grouped on four temperament scales (40 items for novelty seeking; 35 for harm avoidance; 24 for reward dependence; 8 for persistence) and three character scales (44 items for self-directedness; 42 for cooperativeness; 33 for self-transcendence). The total score for each scale is obtained by adding the related items (range of total scores: novelty seeking, 0–40; harm avoidance, 0–35; reward dependence, 0–24; persistence, 0–8; self-directedness, 0–44; cooperativeness, 0–42; self-transcendence 0–33). The higher the total score, the higher the level of the personality dimension described by the scale.

#### Cope Orientation to the Problems Experienced—New Italian Version

The Cope Orientation to Problem Experienced—New Italian Version, COPE-NIV, (31) is a self-report questionnaire that measures coping strategies: the cognitive and behavioral strategies people use to manage stressful situations (32). The COPE-NIV consists of 60 items that describe different coping strategies. Participants are asked to rate each item on a frequency scale from 1 (*I usually don't do this*) to 4 (*I usually do this*). The items are grouped in five essentially independent components: social support (12 items), avoidance strategies (16 items), positive attitude (12 items), problem solving (12 items), and turning to religion (8 items). The total score for the subscales is obtained by adding the corresponding item scores. The higher the total subscale score, the higher the frequency in adopting the coping strategy described by that subscale.

#### Behavioral Inhibition System and Behavioral Activation System Scale

The Behavioral Inhibition and Behavioral Activation Scale, BIS/BAS, (33) is a self-report questionnaire that measures an individual's sensitivity to behavioral inhibition and behavioral activation systems. The behavioral inhibition system (BIS) mediates responses to potential punishment and suppresses behavior that is expected to lead to threat, punishment or non-reward (34). Conversely, the behavioral activation or approach system mediates responses to rewards expectancy (34) and facilitates behavior that brings the person closer to expected rewards. The BIS/BAS scale consists of 24 items grouped in four subscales: BIS (7 items), BAS Drive (4 items), BAS Fun seeking (4 items), and BAS Reward responsiveness (5 items). Four items serve as control. Participants rate how much each statement describes themselves from 0 (*It does not describe me at all*) to 5 (*It completely describes me*). The total score for each scale is obtained by adding the items of the respective scale; the total BIS score ranges from 1 to 35 and the total BAS score ranges from 1 to 65. The higher the total score, the higher the sensitivity of behavioral inhibition and behavioral activation systems.

#### Life-Orientation Test-Revisited

The Life Orientation Test-revisited, LOT-R, (35) is a self-report measure of dispositional optimism and pessimism. It consists of 10 items: 3 are worded positively; 3 are worded negatively; and 4 are control items. Participants rate their agreement with each item on a 5-point scale from 0 (*strongly disagree*) to 4 (*strongly agree*). The LOT-R total score is obtained by adding all items except the 4 control items. The three negatively worded items are related to pessimism and are reverse scored. The LOT-R total score ranges from 0 to 24. The higher the total score, the higher the degree of optimism (high optimism, 19–24; moderate optimism, 14–18; low optimism, 0–13).

### Data Analyses

Distribution of age and gender was analyzed with an independent sample *t*-test and a chi-square test, respectively. Questionnaire data were first checked for normality by means of the Shapiro–Wilk test (*p* > 0.05). Since the data were not normally distributed, non-parametric tests were used. The Friedman test was used to compare questionnaire scores across time points (T0, T1, T2, T3) separately for each group (experimental and control). *Post-hoc* comparisons were performed using the Wilcoxon signed-rank test. Bonferroni correction was applied and the adjusted critical level of significance for multiple comparisons was set at *p* < 0.017. The Mann–Whitney *U*-test was used to compare questionnaire scores between-groups. *P* <0.05 were considered statistically significant. Spearman correlations were used to explore the relationship between mood and sleep quality, separately for each group. Mean and standard deviation of questionnaire data are reported in Tables S1, S2.

## Results

Nineteen participants assumed probiotics and made up the experimental group (7 women, mean age ± SD, 21.47 ± 2.22 years); 19 participants received placebo and made up the control group (6 women, mean age, 22.53 ± 3.64 years). One subject from the experimental group and four subjects from the control group did not complete the study because they underwent therapeutic interventions with antibiotics. The final study sample was 33 subjects: 18 in the experimental group (7 women, mean age, 21.61 ± 2.2 years) and 15 in the control group (5 women, mean age, 21.67 ± 2.19 years). The two groups did not differ by age [*t*_(31)_ = −0.115, *p* = 0.909] or gender distribution [$\chi_{\left(1\right)}^{2}$ = 0.109, *p* = 0.741].

Recruitment started 28 November 2016 and follow-up finished 15 June 2017.

### Mood-Related Aspects

#### Leiden Index of Depression Sensitivity-Revised Test

**Figure 1:**
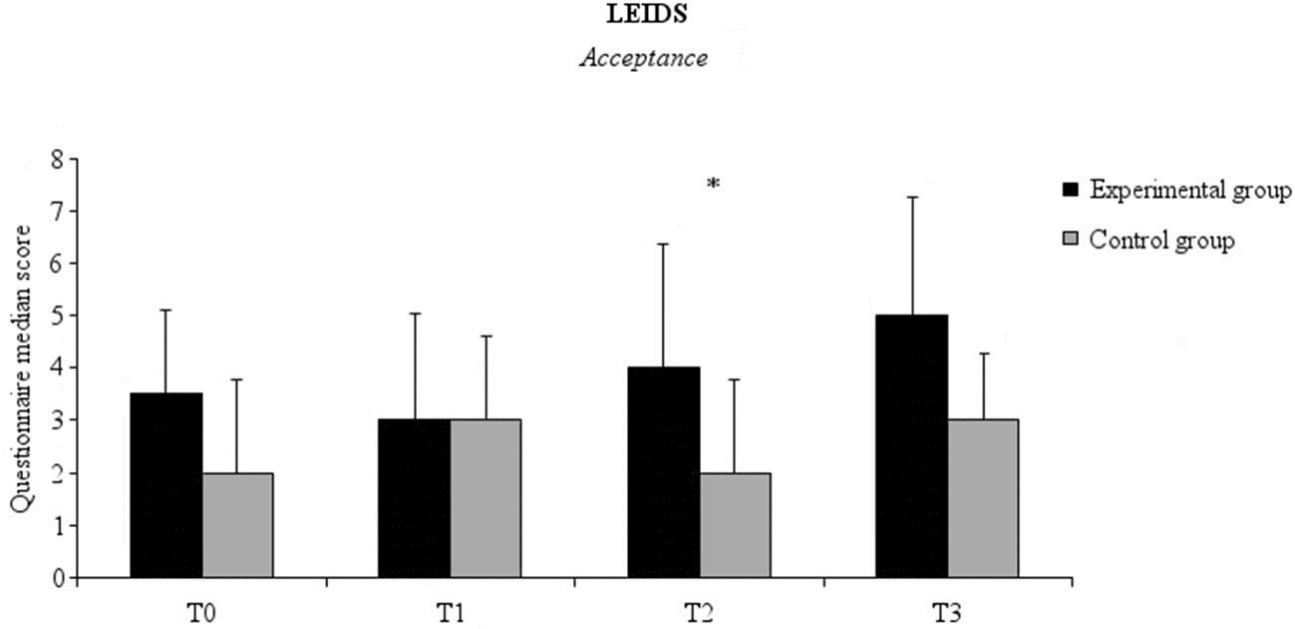
Median LEIDS—R score, Acceptance subscale for the experimental (black columns) and the control (gray columns) groups at all-time points. Error bars represent 95% Confidence Interval. Asterisks indicate between-groups significant differences (*p* < 0.05).

No significant time effect on the total score and subscale scores of the LEIDS-R was noted for the experimental and the control group. There was a significant between-group difference in the *acceptance* subscale scores at 6 weeks (T2). This difference was due to the higher scores recorded in the experimental group (mean ± SD, 5.33 ± 5.11) as compared to the control group (mean ± SD, 2.93 ± 3.51) (*Z* = −2.162, *p* = 0.031, effect size = −0.376) (Figure 1).

#### State Trait Anxiety Inventory

No significant time effect on STAI scores in the experimental and the control group was found: STAI-Y1 (state anxiety) (experimental group, *p* = 0.612; control group, *p* = 0.412) and STAI-Y2 (trait anxiety) (experimental group, *p* = 0.161; control group, *p* = 0.085). Moreover, there were no between-group differences in STAI-Y1 (all time points, *p* > 0.218) and STAI-Y2 (all time points, *p* > 0.436). The state and trait dimensions of anxiety remained constant in both groups at all four time points, discarding a potential effect of probiotics intake on both anxiety components.

#### Beck Depression Inventory

No significant time effect on BDI-2 scores in the experimental (*p* = 0.137) and the control group (*p* = 0.409) was found; there were no between-group differences at any of the four time points (all time points, *p* > 0.383). Probiotics intake had no effect on depressive symptoms as assessed by the BDI-2.

#### Profile of Mood State

**Figure 2:**
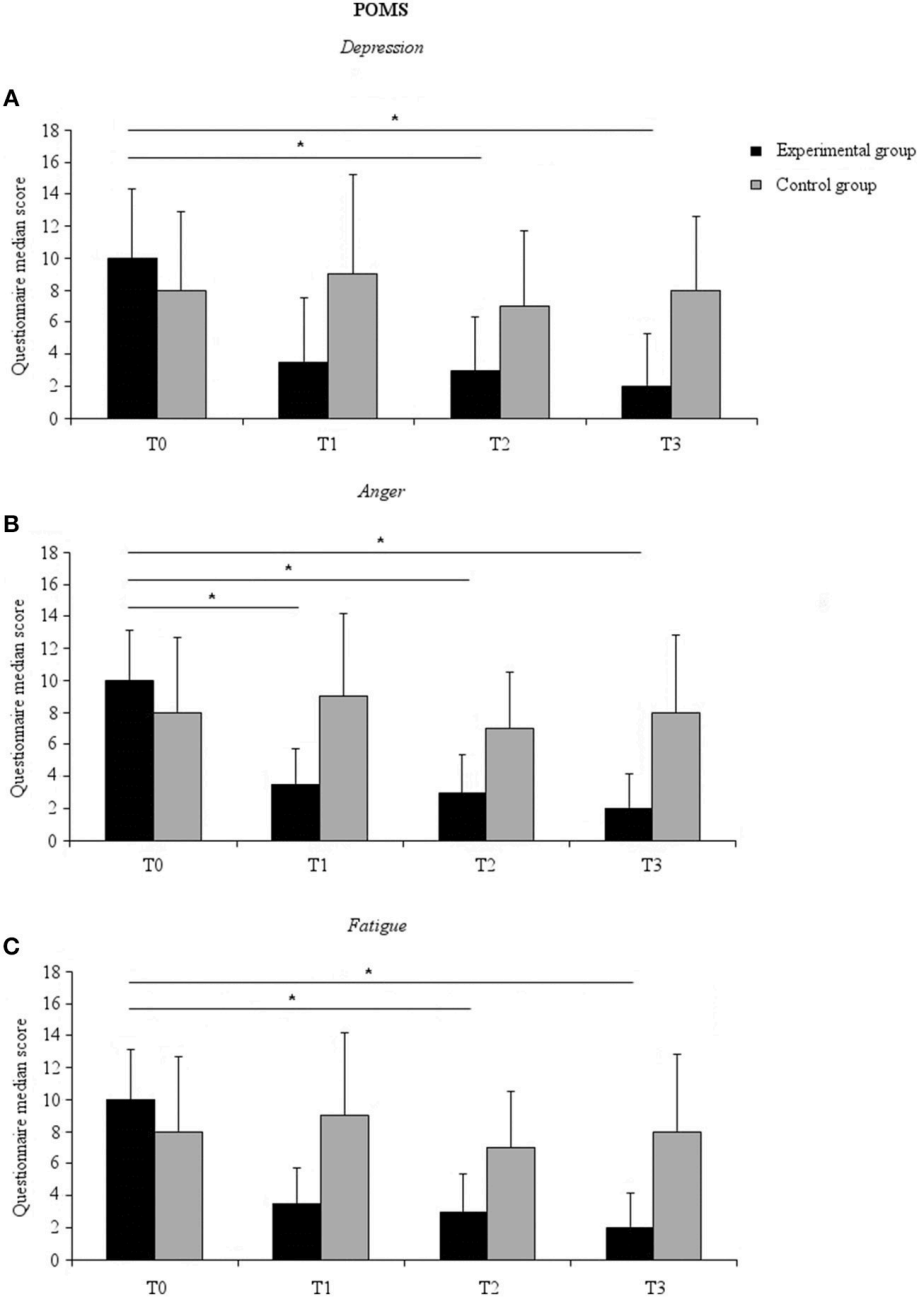
Median POMS subscale score for the experimental (black columns) and the control (gray columns) groups at all-time points. **(A)** Depression subscale; **(B)** Anger subscale; **(C)** Fatigue subscale. Error bars represent 95% confidence interval. Asterisks indicate within-groups significant differences (Bonferroni corrected *p* < 0.017).

A significant time effect on the *depression* subscale scores for the experimental group [χ^2^_(3)_ = 12.43, *p* = 0.006] was found. *Post-hoc* comparisons with Wilcoxon signed rank test (after Bonferroni correction, *p* < 0.017) showed that this result was due to lower scores after 6 weeks of treatment (T2) (mean ± SD, 6.22 ± 7.26) compared to baseline (T0) (mean ± SD, 11.00 ± 9.45) (*Z* = −2.596, *p* = 0.009, effect size = −0.433). Moreover, the effect was maintained after 3 weeks of washout, as revealed by a significant difference between T3 (mean ± SD, 5.67 ± 7.15) and T0 (*Z* = −2.452, *p* = 0.014, effect size = −0.409). These results suggest that the experimental group experienced a reduction in depressive mood state and that this effect remained nearly stable after 3 weeks of washout (Figure 2A).

A significant time effect [χ^2^_(3)_ = 17.52, *p* = 0.001] was also found on the *anger-hostility* scale scores for the experimental group. *Post-hoc* comparisons showed that this effect was due to lower scores after 3 weeks of probiotics intake (T1) (mean ± SD, 6.56 ± 4.87) compared to baseline (T0) (mean ± SD, 10.39 ± 6.79) (*Z* = −2.695, *p* = 0.007, effect size = −0.449), and after 6 weeks of probiotics intake (T2) (mean ± SD, 6.78 ± 5.11) compared to baseline (*Z* = −2.411, *p* = 0.016, effect size = −0.402), as well as after 3 weeks of washout (T3) (mean ± SD, 5.28 ± 4.66) compared to baseline (*Z* = −2.921, *p* = 0.003, effect size = −0.487) (Figure 2B).

A significant time effect was noted for the *fatigue* subscale scores [χ^2^_(3)_ = 11.75, *p* = 0.008]. This effect was due to a lower score at T2 (mean ± SD, 7.06 ± 4.24) compared to T0 (mean ± SD, 8.89 ± 4.30) (*Z* = −2.802, *p* = 0.005, effect size = −0.467) and at T3 (mean ± SD, 5.33 ± 4.12) compared to T0 (*Z* = −2.556, *p* = 0.011, effect size = −0.426) (Figure 2C). A significant time effect was also observed for the subscale *confusion* [χ^2^_(3)_ = 14.45, *p* = 0.003]. This effect was not further confirmed at *post-hoc* analysis, however (*p* > 0.022 for all comparisons). No significant time effect on any of the subscales for the control group. Finally, no between-group differences were found (*p* > 0.079 all-time points and subscales).

### Sleep Quality

#### Pittsburgh Sleep Quality Index

**Figure 3:**
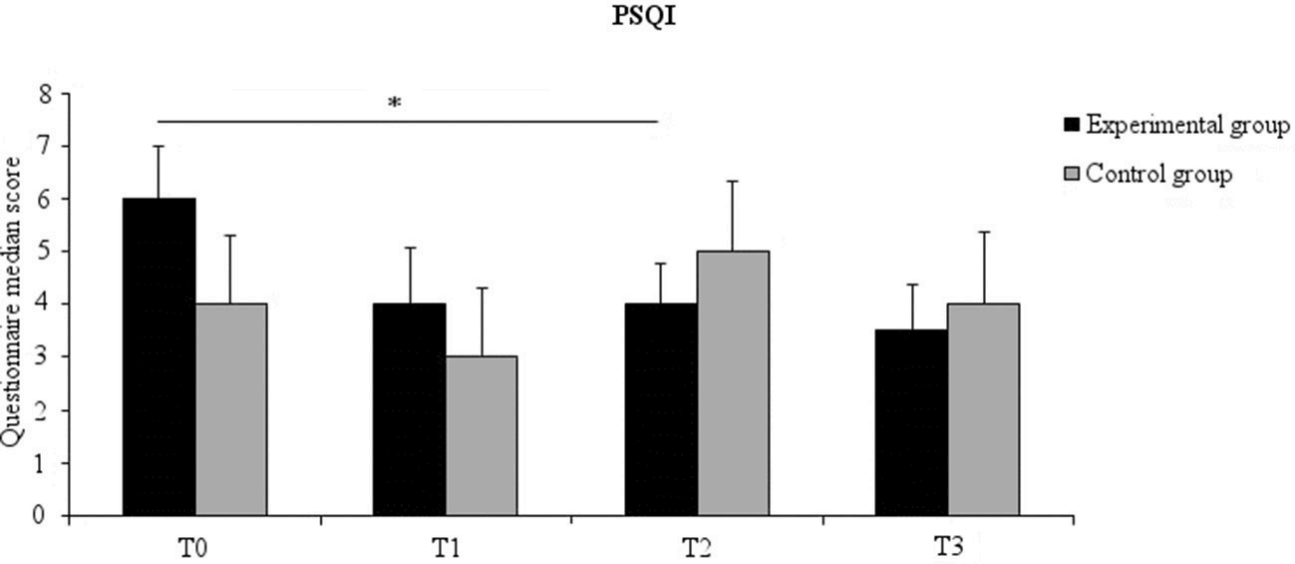
Median PSQI score for the experimental (black columns) and the control (gray columns) groups at all time points. Error bars represent 95% confidence interval. Asterisks indicate within-groups significant differences (Bonferroni corrected *p* < 0.017).

A significant time effect in the experimental group [χ^2^_(3)_ = 12.16, *p* = 0.007] was found. *Post-hoc* comparisons showed that this effect was due to lower scores after 6 weeks of probiotics intake (T2) (mean ± SD, 4.00 ± 1.64) compared to baseline (T0) (mean ± SD, 5.61 ± 2.17) (*Z* = −2.820, *p* = 0.005, effect size = −0.470) (Figure 3). No significant time effect was noted for the control group nor were between-group differences found.

### Correlations Between Mood and Sleep Quality

In the experimental group significant positive correlations were found between PSQI scores and STAI Y2 (T0, *r* = 0.716, *p* = 0.001; T1, *r* = 0.623 *p* = 0.006) and BDI2 (T2, *r* = 0.634, *p* = 0.005). PSQI positively correlated also with several scales of LEIDS-r: Hopelessness (T3, *r* = 0.502, *p* = 0.034); Acceptance (T3, *r* = 0.834, *p* < 0.001); Aggression (T0, *r* = 0.544, *p* = 0.020); Risk aversion (T0, *r* = 0.472, *p* = 0.048). Significant correlations were also found with different scales of POMS as well. Precisely, PSQI positively correlated with Tension (T0, *r* = 0.628, *p* = 0.005; T2, *r* = 0.643, *p* = 0.004), Depression (T0, *r* = 0.709, *p* = 0.001; T2, *r* = 0.549, *p* = 0.018), Anger (T0, *r* = 0.829, *p* < 0.001; T2, *r* = 0.679, *p* = 0.002), Fatigue (T0, *r* = 0.595, *p* = 0.009; T2, *r* = 0.560, *p* = 0.016), and Confusion (T0, *r* = 0.595, *p* = 0.009) subscales. Negative correlations, instead, were found with the Vigor subscale (T0, *r* = −0.545, *p* = 0.019; T3, *r* = −0.507, *p* = 0.032). As stated before, the higher the scores at PSQI, the lower the sleep quality. Thus, positive correlations with measures of anxiety (i.e., STAI Y2) and depression (i.e., BDI2) suggest that the lower the sleep quality, the higher the anxiety and depression. Similarly, poor sleep quality is related to higher hopelessness, acceptance, aggression, risk aversion as measured by the LEIDS-r subscales, and tension, depressive mood state, anger, fatigue, and confusions as measured by the POMS subscales. Conversely, the negative correlation with the Vigor subscale suggests that the better the sleep quality, the higher the level of vigor experimented by participants. In the control group PSQI positively correlated with STAI-Y1 (T1, *r* = 0.550, *p* = 0.034; T2, *r* = 0.638, *p* = 0.010), STAI-Y2 (T1, *r* = 0.707, *p* = 0.003; T2, *r* = 0.738, *p* = 0.002) and BDI2 (T1, *r* = 0.710, *p* = 0.003). Significant correlations were also observed for several subscales of POMS. More precisely, sleep quality positively correlated with Tension (T0, *r* = 0.537, *p* = 0.039; T1, *r* = 0.601, *p* = 0.018; T2, *r* = 0.595, *p* = 0.019), Anger (T1, *r* = 0.615, *p* = 0.015), Fatigue (T2, *r* = 0.677, *p* = 0.006), and Confusion (T1, *r* = 0.694, *p* = 0.004) subscales, while negatively correlated with the Vigor subscale (T1, *r* = −0.771, *p* = 0.001; T2, *r* = −0.537, *p* = 0.039). These results suggest that a poor sleep quality relates to higher anxiety, depression, tension, anger, fatigue, and confusion. Conversely, a good sleep quality relates to higher vigor.

### Personality-Related Aspects

#### Temperament and Character Inventory

No significant time effect on any of the TCI subscale scores in either the experimental or the control group was found. Significant between-group differences were found for four subscale scores at different time points. There was a significant group effect for the *novelty seeking* subscale score only at T3 (*Z* = −2.209, *p* = 0.027, effect size = −0.385), where the scores were higher for the experimental group. This finding might suggest that at the last assessment the participants who had taken probiotics for 6 weeks were more prone to look for new experiences; in other words, they may have been more activated by novel stimuli as compared to the controls. In addition, the *persistence* subscale scores differed between the groups; the difference was due to lower scores for the experimental group at all-time points (T0, *Z* = −3.127, *p* = 0.002, effect size = −0.544; T1, *Z* = −2.906, *p* = 0.004, effect size = −0.506; T2, *Z* = −2.948, *p* = 0.003, effect size = −0.513; T3, *Z* = −2.820, *p* = 0.005, effect size = −0.491). This means that starting from baseline, the experimental group was less persistent and that these differences remained constant independent of probiotics intake.

A significant group effect was found for the *self-directedness* subscale scores at T3 (*Z* = −2.497, *p* = 0.013, effect size = −0.435). This difference was due to the lower scores for the experimental group, suggesting that at 3 weeks since their last intake of the probiotic the experimental group was less self-directed than the controls.

Finally, there was a significant between-group difference in *cooperativeness* subscales scores at T2 (*Z* = −2.233, *p* = 0.026, effect size = −0.389), with lower scores noted for the experimental group. This finding might suggest that after 6 weeks of probiotics intake, the participants who had taken the probiotics were more self-focused than those who did not.

#### Coping Orientation to Problem Experienced

No significant time effect on any of the COPE subscale scores in either the experimental or the control group was noted. Significant between-group differences were found for the *Avoidance Strategies* subscale scores at T2 (*Z* = −2.590, *p* = 0.010, effect size = −0.451) and T3 (*Z* = −2.728, *p* = 0.006, effect size = −0.475), where the scores were higher for the experimental group, suggesting that they were more apt than the control group to use avoidance strategies after 6 weeks of probiotics intake and after 3 weeks of washout.

Moreover, there was a significant between-group difference in the *Positive Attitude* subscale scores at T1 (*Z* = 2.412, *p* = 0.016, effect size = −0.420) due to lower scores for the experimental group, suggesting that after 3 weeks of probiotics intake, the experimental group had a lower positive attitude toward problem solving than the controls. This difference was not observed at the other time points, however.

#### Behavioral Inhibition System and Behavioral Activation System Scale

No differences between time points for either group nor between-group differences were found. These results suggest that probiotics intake did not affect the inhibition or the activation system.

#### Life Orientation Test-Revisited Test (LOT-R)

No significant time effect on either group was found. Moreover, no differences between-groups were found. These results suggest that probiotics intake did not influence the level of optimism in the experimental group.

## Discussion

With this exploratory study we investigated the effect of probiotics intake on different aspects of mood and dimensions of personality in healthy individuals. Overall, probiotics intake was noted to exert a positive effect on depressive mood state and sleep quality. This effect was specific for the experimental group, thus ruling out confounding factors like learning, expectations or maturation. We suggest, with caution, that these findings provide new support to the notion that probiotics may exert a beneficial psychological influence.

### Effect of Probiotics on Mood-Related Aspects

Previous studies in non-clinical samples have demonstrated that multispecies probiotics have positive effects on mood (12, 36, 37). These studies focused mainly on depression and anxiety, whereas we extend previous findings with a more comprehensive assessment of these functions during and after 6 weeks of probiotics intake.

Among the different indexes of cognitive reactivity to sad mood assessed by means of the LEIDS-R, the scores for *acceptance* were found to be higher in the experimental group than the control group after 6 weeks of probiotics intake. Acceptance is an emotion regulation strategy that facilitates recovery from depression in clinical populations (38). We speculate that increasing acceptance might protect healthy individuals from developing depressive mood.

Like Steenbergen et al. (12), we found no significant differences between-groups or across time points for depression scores as assessed by the BDI. This was not surprising since our participants all demonstrated being below the clinically relevant score for depression, suggesting that they did not have depressive symptoms. Nonetheless, using the POMS we were able to detect specific changes in positive and negative mood in healthy individuals. In line with previous studies (12, 36, 37), we found a reduction in depressive mood state in the group that received the probiotics. The *depression* subscale score was lower after 6 weeks of probiotics intake, suggesting that the experimental group benefited from probiotics intake. Interestingly, this positive effect was maintained after 3 weeks of washout, suggesting a long-term effect. Moreover, we found a reduction in anger as measured by the *anger* subscale of the POMS. This reduction appeared at the 3-week assessment and was consistent across other time points. Hence, the scores for anger were lower in the experimental group not only during the 6-week period of probiotics intake but also after 3 weeks of washout. The sense of *fatigue* was also lower in the experimental group at 6 weeks of probiotics intake and continued after the 3 weeks of washout. Since no between-group differences were detected, caution is needed in interpreting these findings. Nonetheless, we suggest that, overall, these findings hint at a beneficial effect of probiotics intake in the experimental group.

No significant differences were found for STAI scores, thus suggesting no changes in state and trait anxiety as assessed by STAI-Y1 and STAI-Y2. Of note, the STAY-Y1 measures the perceived anxiety level at a precise time point. In our study, this time point corresponded to the assessment sessions. The lack of differences between-groups and across time points may indicate that the study participants were relaxed and did not feel they were under pressure while completing the questionnaires, thus excluding potential biases due to anxiety.

The STAI-Y2, instead, measures a more stable dimension of anxiety, the trait anxiety, i.e., an individual's propensity to habitually give anxious responses. The lack of effect of probiotics on this trait could be explained by the fact that healthy individuals already have low anxiety scores that cannot be further lowered by probiotics intake.

### Effect of Probiotics on Sleep Quality

Although we did not find significant differences between the experimental and control group at the PSQI, the former but not the latter reported an improvement in sleep quality. More precisely, the experimental group participants reported that their sleep quality improved after 6 weeks of probiotics intake. The conclusions that can be drawn from these findings are limited by the lack of between-group differences. At any rate, we suggest that probiotics intake had beneficial effects on sleep quality in experimental group. Recent studies have demonstrated that probiotics ameliorate sleep in patients with chronic fatigue syndrome (39), chronic pain (40), and in stressed medical students (41). Our finding extends previous observations, suggesting that the probiotics mixture we used was effective in improving sleep quality also in a healthy population. Moreover, the improvement in sleep quality fits well with the reduction in depressive mood state, anger, and fatigue we observed in the experimental group. Previous studies demonstrated a strong relationship between sleep quality and mood (15). This was also the case of our study. Correlation analysis indeed, showed that sleep quality correlated with different aspects of mood, like anxiety, depressive symptoms, fatigue, anger, confusion and vigor, and additionally, in the experimental group, with some aspects of cognitive reactivity to sad mood (e.g., aggression, risk aversion, acceptance and hopelessness). Probiotics might have positively influenced mood not only by influencing emotional processes, but also other related aspects such as sleep quality as well.

### Effect of Probiotics on Aspects Related to Personality and its Relationship With Mood

We found no differences in personality traits across time points, suggesting that the two groups remained stable throughout the study. This suggests that personality dimensions are complex and consolidated features that cannot be easily modified over time. Whether the lack of effect was due to the duration of the study (too short) or to the dose of the probiotics (too small) will need to be investigated in a future study.

At certain time points and for specific TCI subscales, however, we found significant between-group differences. For instance, *novelty seeking* scores (i.e., exhilaration in response to novel stimuli) were higher for the experimental than the control group at T3, i.e., after 3 weeks of washout. Caution is warranted when interpreting these findings, however, since the between-group difference in novelty seeking was not consistent across time points. Nonetheless, we suggest that the improvement in mood induced by the probiotics could have facilitated novelty seeking in the experimental group. Support for this hypothesis derives from the evidence that positive emotions trigger exploratory behavior [for a review, see (42)]. Similarly, *self-directedness* and *cooperativeness* subscale scores were significantly different between the two groups only at T3 and at T2, respectively. Once again, because these differences were present only at single time points, it is difficult to draw strong associations with probiotics intake.

Two subscales in the COPE inventory, the *avoidance strategies* and *the positive attitude* subscales, revealed differences between the two groups at more than one time point. We found higher scores on the *avoidance strategies* subscale for the experimental group compared to the control group at T2 (after 6 weeks of probiotics intake) and at T3 (after 3 weeks of washout). This means that the experimental group participants were more likely to adopt strategies based on avoidance (e.g., through denial of stressful situations) when they experienced problems. Taking into account the findings on mood, we speculate that because the experimental group participants felt better, as shown by the reduced depressive mood state, anger and fatigue, they were also more likely to avoid negative thoughts or situations, which induced a positive bias in their responses. In this regard, it has been demonstrated that people who feel in a positive mood are motivated to maintain their positive state (43). For instance, studies on risk-taking behavior demonstrated that positive feelings can accentuate an aversion toward risky options related to a higher probability of loss (43). Of note, avoidance strategies allow to deny problems and related emotions that alter positive mood. In this sense, they might represent the “less risky option” to temporarily prevent loss of a positive state. Following this line of reasoning, we believe that the experimental group participants probably had a higher propensity to adopt avoidance strategies in order to preserve their acquired positive state. This is only speculative and cannot be completely supported by our data; nonetheless, it deserves further investigation.

Also, the *positive attitude* subscale scores were lower for the experimental compared to the control group at T1 (at 3 weeks into the study). Positive attitude is an adaptive coping strategy that allows individuals to positively reinterpret negative situations (44). Hence, a lower score for this subscale suggests that the experimental group participants were temporarily less prone than the controls to positively reinterpret a negative situation. How does this result fit with the amelioration of mood observed in the experimental group? It has been widely demonstrated that positive feelings can bias attention toward mood-congruent stimuli (45, 46). In virtue of this congruency effect, people who feel in a positive mood are apt to attend to positive rather than negative events (46). We speculate that, in virtue of a general positive mood, the experimental group participants subjects did not need to reinterpret negative situations probably because they tended to focus on positive events congruent with their mood rather than on negative events.

#### Limitations of the Study

Some limitations of this study should be acknowledged. For instance, the small sample size and the potential pre-existing group differences with regard to some personality traits (e.g., *persistence* subscale of the TCI) may limit generalizability of the findings. Future studies with larger sample will allow to apply a randomization method that allows to take into account more than one covariate (21). Moreover, due to time constraints, we used only dimensional questionnaires to assess personality, thus limiting our findings. Future investigations with categorical diagnostic tools (e.g., Structured Clinical Interview for DSM-IV, SCID-II) will allow to achieve a more comprehensive view by assessing personality disorders. Despite these limitations, we think that our study indicates that probiotics might help to promote psychological well-being. As one of the possible underlying mechanisms, we speculate that probiotics, by interacting with intestinal microbiota, facilitated the production of precursors of neuroactive substances involved in modulating emotional processing, sleep, and other brain functions (5, 6). Since the current study did not focus on changes in intestinal microbiota following probiotic intake, this is only a speculation based on previous findings (5, 6). Future studies on larger samples are needed to verify this hypothesis and to uncover the biological mechanisms at the basis of these effects by investigating the changes in the intestinal microbiota related to probiotics intake. Moreover, it would be interesting to extend these investigations to clinical populations and verify whether the positive effects of probiotics intake can be observed in such patients. If so, this would suggest the use of probiotics as adjunctive therapy for conditions characterized by altered mood and/or sleep disturbances.

## Concluding Remarks

We did not find significant differences between the experimental and control group neither at mood-related questionnaires, nor at the questionnaire assessing the quality of sleep. Nonetheless, we found changes in mood state and sleep quality across sessions in the experimental and not in the control group. More precisely, our exploratory study shows an improvement across time in different aspects of the profile of mood state, like sad mood, anger, and fatigue only in healthy individuals which took probiotics. Sleep quality also reportedly improved after probiotics intake. Of note, sleep quality strongly relates to mood. More precisely, as suggested by the significant correlations between sleep quality and mood-related questionnaires, the higher the quality of sleep, the better the mood state in the experimental and in the control group. Finally, significant differences between-groups have been observed for some personality-related questionnaires. In this regard, our findings suggest that improving mood by means of probiotics intake might additionally determine changes in cognitive strategies to deal with problems by reducing sensitivity to negative situations, as well as the need to deal with them. Moreover, we speculate that ameliorating mood can differentially affect an individual's predisposition, for instance, by facilitating novelty seeking.

## Author Contributions

MF, GF, and AD conception and design of the research project. MF, GF, AD, and AM organization of the research project. AM and ES data acquisition. AM and MF data analysis. AM, MF, GF, and AD interpretation of the data. AM writing the first draft. MF, GF, ES, MP, AA, and AD revision and comments on the first draft. AM, ES, AD, MP, AA, LM, GF, and MF final approval of the manuscript.

### Conflict of Interest Statement

MP, LM, and AA were employed by company BIOLAB s.r.l. The remaining authors declare that the research was conducted in the absence of any commercial or financial relationships that could be construed as a potential conflict of interest.
