# Supplementary material for: Effects of Probiotics on Cognitive Reactivity, Mood, and Sleep Quality
Source: Front Psychiatry. 2019 Mar 27;10:164. doi: 10.3389/fpsyt.2019.00164 (PMC6445894; doi:10.3389/fpsyt.2019.00164)
Supplement: Supplementary file 1 [file Table_1.docx]

Supplementary Material

**Effects of probiotics on cognitive reactivity, mood and sleep quality**

Marotta A^1,2*^, Sarno E^3¶^, Del Casale A^4¶^, Pane M^5^, Mogna L^5^, Amoruso A^5^, Felis GE^3^, Fiorio M^1*^

*** Correspondence:** Angela Marotta: angela.marotta@univr.it

# Supplementary Table 1

|  | Experimental group | | | | Control group | | | |
| --- | --- | --- | --- | --- | --- | --- | --- | --- |
|  | Assessment sessions | | | | Assessment sessions | | | |
|  | T0 | T1 | T2 | T3 | T0 | T1 | T2 | T3 |
| **LEIDS-R** |  |  |  |  |  |  |  |  |
| Hopelessness | 5.44 (5.16) | 5.06 (5.82) | 5.00  (4.68) | 6.11 (5.55) | 3.33 (3.35) | 3.40 (3.22) | 2.93 (3.71) | 3.33 (3.09) |
| Acceptance | 4.78 (3.47) | 4.22 (4.41) | 5.33 (5.11) | 5.83 (4.94) | 2.80 (3.49) | 3.33 (3.20) | 2.93 (3.51) | 2.93 (2.49) |
| Aggression | 8.39 (4.55) | 9.00 (4.96) | 8.22 (4.66) | 8.94 (5.24) | 7.53 (3.70) | 5.93 (3.53) | 5.20 (2.57) | 5.47 (4.32) |
| Control | 7.72 (3.12) | 7.61 (5.23) | 7.72 (3.88) | 8.06 (3.28) | 7.07 (4.38) | 6.87 (4.81) | 6.73 (4.18) | 6.80 (4.14) |
| Risk aversion | 10.22 (3.10) | 9.39 (3.94) | 9.78 (3.59) | 10.33 (3.82) | 9.00 (2.75) | 9.27 (3.61) | 8.53 (6.64) | 7.87 (4.26) |
| Rumination | 11.61 (3.85) | 10 (5.20) | 11.17 (4.63) | 10.06 (4.19) | 12.20 (5.88) | 10.53 (6.27) | 9.93 (4.77) | 10.33 (5.65) |
|  |  |  |  |  |  |  |  |  |
| **STAI** |  |  |  |  |  |  |  |  |
| Form - Y1 | 36.67 (8.40) | 34.39 (7.48) | 36.44 (9.12) | 33.83 (7.12) | 33.87 (5.21) | 38.93 (13.29) | 36.00 (11.72) | 38.13 (12.20) |
| Form - Y2 | 41.50 (7.02) | 40.61 (7.48) | 37.39 (8.72) | 37.61 (8.49) | 41.60 (9.08) | 42.40 (10.63) | 40.20 (11.38) | 39.47 (8.52) |
|  |  |  |  |  |  |  |  |  |
| **BDI-2** | 9.22 (9.49) | 5.83 (5.08) | 5.50  (5.54) | 4.89 (4.21) | 8.33 (5.21) | 8.80 (9.03) | 7.47 (7.35) | 7.13 (6.53) |
|  |  |  |  |  |  |  |  |  |
| **POMS** |  |  |  |  |  |  |  |  |
| Tension | 8.61 (5.34) | 6.00 (4.07) | 5.89 (3.56) | 5.44 (5.59) | 12.40 (6.73) | 8.47 (5.89) | 9.40 (6.24) | 8.47 (5.94) |
| Depression | 11.00 (9.45) | 7.28 (8.80) | 6.22 (7.26) | 5.67 (7.15) | 9.80 (9.64) | 11.80 (12.34) | 8.53 (9.33) | 8.73 (9.18) |
| Anger | 10.39 (6.79) | 6.56 (4.87) | 6.78 (5.11) | 5.28 (4.66) | 11.40 (9.21) | 9.13 (10.24) | 9.60 (6.98) | 8.53 (9.49) |
| Vigor | 16.78 (3.89) | 18.06 (4.84) | 16.17 (5.70) | 17.61 (6.25) | 15.40 (7.39) | 17.00 (7.19) | 15.00 (7.31) | 14.87 (8.10) |
| Fatigue | 8.89 (4.30) | 7.06 (4.24) | 5.28 (3.97) | 5.33 (4.12) | 10.53 (5.01) | 8.40 (5.18) | 7.87 (4.58) | 7.20 (5.45) |
| Confusion | 9.50 (3.47) | 8.78 (4.02) | 7.33 (4.83) | 6.89 (5.14) | 10.33 (5.96) | 11.13 (6.17) | 10.13 (6.64) | 7.80 (3.97) |
|  |  |  |  |  |  |  |  |  |
| **PSQI** | 5.61 (2.17) | 4.67 (2.35) | 4.00 (1.64) | 4.22 (1.86) | 4.67 (2.61) | 3.87 (2.59) | 4.33 (2.66) | 4.13 (2.70) |

**Supplementary Table 1.** Mean (and standard deviation) of the questionnaires measuring mood-related aspects and sleep quality at baseline (T0), 3 weeks (T1) and 6 weeks (T2) after the first intake and at 3 weeks of washout (T3) in the experimental and control group.
