# Supplementary material for: Effects of Probiotics on Cognitive Reactivity, Mood, and Sleep Quality
Source: Front Psychiatry. 2019 Mar 27;10:164. doi: 10.3389/fpsyt.2019.00164 (PMC6445894; doi:10.3389/fpsyt.2019.00164)
Supplement: Supplementary file 2 [file Table_2.docx]

Supplementary Material

**Effects of probiotics on cognitive reactivity, mood and sleep quality**

Marotta A^1,2*^, Sarno E^3¶^, Del Casale A^4¶^, Pane M^5^, Mogna L^5^, Amoruso A^5^, Felis GE^3^, Fiorio M^1*^

*** Correspondence:** Angela Marotta: angela.marotta@univr.it

# Supplementary Table 2

**Supplementary Table 2.** Mean (and standard deviation) of questionnaires measuring personality-related aspects at baseline (T0), 3 weeks (T1) and 6 weeks (T2) after the first intake and at 3 weeks of washout (T3) in the experimental and control group.

|  | Experimental group | | | | Control group | | | |
| --- | --- | --- | --- | --- | --- | --- | --- | --- |
|  | Assessment sessions | | | | Assessment sessions | | | |
|  | T0 | T1 | T2 | T3 | T0 | T1 | T2 | T3 |
| **TCI** |  |  |  |  |  |  |  |  |
| Novelty seeking | 21.39 (4.33) | 22.50 (5.14) | 22.44 (5.39) | 22.22 (5.80) | 17.47 (5.10) | 18.80 (6.22) | 18.53 (7.06) | 17.60 (4.95) |
| Harm avoidance | 14.39 (5.94) | 14.94 (7.57) | 13.61 (7.40) | 12.72 (7.27) | 16.27 (6.47) | 14.73 (8.28) | 14.20 (8.16) | 14.33 (8.93) |
| Reward dependence | 13.83 (4.41) | 14.39 (4.65) | 13.61 (4.41) | 13.11 (3.89) | 15.47 (4.47) | 16.00 (4.60) | 15.53 (3.93) | 15.13 (4.45) |
| Persistence | 4.00 (1.94) | 3.94 (1.95) | 3.72 (1.71) | 4.17 (1.76) | 5.93 (1.10) | 5.93 (1.44) | 5.73 (1.67) | 6.00 (1.65) |
| Self-directedness | 25.89 (7.51) | 24.44 (6.78) | 24.94 (7.25) | 23.61 (8.41) | 29.00 (7.13) | 28.73 (8.58) | 30.60 (7.95) | 31.33 (7.35) |
| Cooperativeness | 29.39 (7.54) | 29.78 (6.90) | 29.83 (6.25) | 30.39 (7.07) | 33.00 (5.13) | 33.67 (5.14) | 34.20 (4.23) | 34.13 (4.41) |
| Self-trascendence | 12.11 (4.44) | 13.28 (5.29) | 10.72 (5.28) | 11.89 (5.72) | 13.60 (6.62) | 14.60 (7.57) | 13.87 (7.89) | 13.00 (8.26) |
| **COPE** |  |  |  |  |  |  |  |  |
| Social support | 30.33 (6.99) | 30.28 (8.92) | 30.67 (7.86) | 30.94 (9.08) | 32.07 (11.30) | 33.87 (10.69) | 31.47 (10.39) | 32.00 (10.51) |
| Avoidance strategies | 29.00 (6.02) | 28.61 (7.22) | 28.72 (7.60) | 29.06 (7.06) | 22.47 (3.83) | 24.13 (4.90) | 22.73 (4.70) | 22.87 (5.01) |
| Positive attitude | 32.94 (3.39) | 30.78 (4.18) | 32.11 (5.04) | 31.89 (3.27) | 36.33 (4.62) | 35.53 (6.06) | 34.67 (5.16) | 34.00 (5.24) |
| Problem solving | 31.06 (4.49) | 30.94 (4.71) | 31.28 (6.44) | 30.39 (6.14) | 35.20 (4.69) | 33.00 (6.36) | 32.07 (7.07) | 31.73 (6.35) |
| Turning to religion | 16.50 (3.73) | 16.11 (3.50) | 16.22 (4.25) | 15.72 (3.34) | 18.20 (5.56) | 18.13 (5.03) | 17.93 (5.54) | 17.47 (5.53) |
| **BIS/BAS** |  |  |  |  |  |  |  |  |
| BIS | 20.83 (3.22) | 19.89 (3.77) | 20.00 (3.61) | 20.89 (3.27) | 21.20 (3.55) | 21.73 (3.39) | 20.53 (2.88) | 21.00 (2.56) |
| BAS – Drive | 13.22 (3.19) | 13.83 (3.15) | 13.72 (2.82) | 14.00 (2.57) | 13.07 (2.96) | 13.27 (2.31) | 12.93 (2.74) | 13.60 (2.92) |
| BAS – Fun seeking | 13.83 (3.29) | 13.67 (3.24) | 14.28 (3.21) | 14.06 (3.06) | 12.60 (3.46) | 12.53 (3.91) | 13.13 (4.05) | 13.27 (3.51) |
| BAS- Reward responsiveness | 20.94 (2.82) | 20.61 (2.95) | 20.83 (3.40) | 20.94 (2.84) | 21.27 (3.20) | 21.40 (2.95) | 20.93 (2.99) | 21.33 (3.44) |
|  |  |  |  |  |  |  |  |  |
| **LOT-r** | 14.06 (4.65) | 14.72 (5.10) | 16.17 (3.67) | 16.44 (4.36) | 15.47 (4.07) | 16.27 (5.31) | 15.40 (3.87) | 16.13 (5.22) |
